# Persistent below-knee great saphenous vein reflux after above-knee endovenous laser ablation with 1470-nm laser: a prospective study

**DOI:** 10.1590/1677-5449.001516

**Published:** 2016

**Authors:** Walter Junior Boim de Araujo, Jorge Rufino Ribas Timi, Carlos Seme Nejm, Fabiano Luiz Erzinger, Filipe Carlos Caron

**Affiliations:** 1 Universidade Federal do Paraná – UFPR, Departamento de Cirurgia, Curitiba, PR, Brazil.

**Keywords:** varicose veins, laser therapy, Doppler ultrasonography, ablation techniques, varizes, terapia a laser, ultrassonografia Doppler, técnicas de ablação

## Abstract

**Background:**

In endovenous laser ablation (EVLA), the great saphenous vein (GSV) is usually ablated from the knee to the groin, with no treatment of the below-knee segment regardless of its reflux status. However, persistent below-knee GSV reflux appears to be responsible for residual varicosities and symptoms of venous disease.

**Objectives:**

To evaluate clinical and duplex ultrasound (DUS) outcomes of the below-knee segment of the GSV after above-knee EVLA associated with conventional surgical treatment of varicosities and incompetent perforating veins.

**Methods:**

Thirty-six patients (59 GSVs) were distributed into 2 groups, a control group (26 GSVs with normal below-knee flow on DUS) and a test group (33 GSVs with below-knee reflux). Above-knee EVLA was performed with a 1470-nm bare-fiber diode laser and supplemented with phlebectomies of varicose tributaries and insufficient perforating-communicating veins through mini-incisions. Follow-up DUS, clinical evaluation using the venous clinical severity score (VCSS), and evaluation of complications were performed at 3-5 days after the procedure and at 1, 6, and 12 months.

**Results:**

Mean patient age was 45 years, and 31 patients were women (86.12%). VCSS improved in both groups. Most patients in the test group exhibited normalization of reflux, with normal flow at the beginning of follow-up (88.33% of GSVs at 3-5 days and 70% at 1 month). However, in many of these patients reflux eventually returned (56.67% of GSVs at 6 months and 70% at 1 year).

**Conclusions:**

These data suggest that reflux in the below-knee segment of the GSV was not influenced by the treatment performed.

## Resumo

**Contexto:**

A termoablação da veia safena magna com laser (em inglês, *endovenous laser therapy* – EVLA) geralmente é realizada do joelho até a virilha, sem tratamento do segmento abaixo do joelho, independentemente do seu status de refluxo. Entretanto, a persistência de refluxo da veia safena magna (VS.M) na perna parece ser responsável por varizes residuais e sintomas da doença venosa.

**Objetivos:**

Avaliar a evolução clínica e os resultados do eco-Doppler da VS.M na perna após EVLA na coxa associada ao tratamento cirúrgico convencional de varizes e veias perfurantes incompetentes.

**Métodos:**

Trinta e seis pacientes (59 VS.Ms) foram divididos em dois grupos: grupo-controle (26 VS.Ms com fluxo normal na perna ao eco-Doppler) e grupo-teste (33 VS.Ms com refluxo na perna). EVLA na coxa foi realizada com laser 1470 nm com fibra nua, associada a flebectomia das veias tributárias e perfurantes-comunicantes insuficientes através de mini-incisões. Acompanhamento com eco-Doppler, avaliação clínica pelo escore de gravidade clínica venosa (em inglês, *venous clinical severity score* – VCSS) e avaliação das complicações foram realizados 3-5 dias após o procedimento e em 1, 6 e 12 meses.

**Resultados:**

A idade média dos pacientes era de 45 anos, e 31 eram mulheres (86,12%). Os dois grupos apresentaram melhora no VCSS. A maioria do grupo-teste apresentou normalização do refluxo, com fluxo normal no início do acompanhamento (88,33% das VS.Ms em 3-5 dias e 70% em 1 mês). Porém, esses pacientes evoluíram com retorno do refluxo (56,67% das VS.Ms em 6 meses e 70% em 1 ano).

**Conclusões:**

Esses dados sugerem que o refluxo da VS.M na perna não foi influenciado pelo tratamento realizado.

## INTRODUCTION

Superficial venous insufficiency can produce a wide variety of signs and symptoms, which in the past were mainly attributed to deep venous disease. Saphenofemoral junction (SFJ) incompetence associated with great saphenous vein (GSV) reflux is the most common cause of varicose veins and chronic venous insufficiency.^1^

The more extensive the anatomic extent of reflux, the higher the incidence of signs and symptoms. It has been reported that reflux confined to the below-knee segment of the GSV is associated with a higher incidence of signs and symptoms than reflux in the above-knee segment.^1^

Conventional surgical treatment of varicose veins involves elimination or reduction of venous hypertension by high ligation of the SFJ and subsequent stripping of the GSV combined with avulsion of visible varicosities (phlebectomy).^2^ However, considerable morbidity and patient dissatisfaction associated with surgical treatment have prompted development of alternative techniques.^3^ One minimally invasive alternative to surgery is endovenous treatment of GSV reflux using thermal damage to promote occlusion of the vein, with success rates ranging from 88 to 100% of limbs.^4^

In endovenous laser ablation (EVLA), as originally described, the GSV is ablated from the knee to the groin with no treatment of the below-knee segment regardless of its reflux status.^5^ However, persistent below-knee GSV reflux appears to be responsible for residual varicosities and residual symptoms of venous disease, suggesting that EVLA of the below-knee segment of the GSV with reflux may be more effective in both respects. However, further studies are required to test this hypothesis.^6^

The aims of the current study were to evaluate duplex ultrasound (DUS) outcomes following above-knee EVLA of the GSV with 1470-nm laser supplemented with conventional surgical treatment of varicosities and incompetent perforating veins and to evaluate clinical outcomes and complications in patients who underwent the proposed treatment regimen.

## MATERIALS AND METHODS

This prospective cohort study was conducted at a tertiary care teaching hospital located in Curitiba, southern Brazil. The study was approved by the institution’s Research Ethics Committee (protocol number 07643012.2.0000.0096) and conducted in accordance with the international ethical standards set out in the Declaration of Helsinki.

Participants were recruited from among patients receiving care at our institution from January 2013 to December 2014. Eligible participants were all patients aged 18 years or over, of both sexes, who had been diagnosed with unilateral or bilateral varicose veins of the lower extremities, with clinical class C2-C6 disease according to the Clinical-Etiology-Anatomy-Pathophysiology (CEAP) classification, and who had been referred for surgical treatment. Exclusion criteria were previous history of superficial and/or deep vein thrombosis, concomitant peripheral arterial disease, difficulty walking, pregnancy, breastfeeding, or previous surgical treatment of varicose veins. Written informed consent was obtained from all individual participants included in the study.

**Figure 1:**
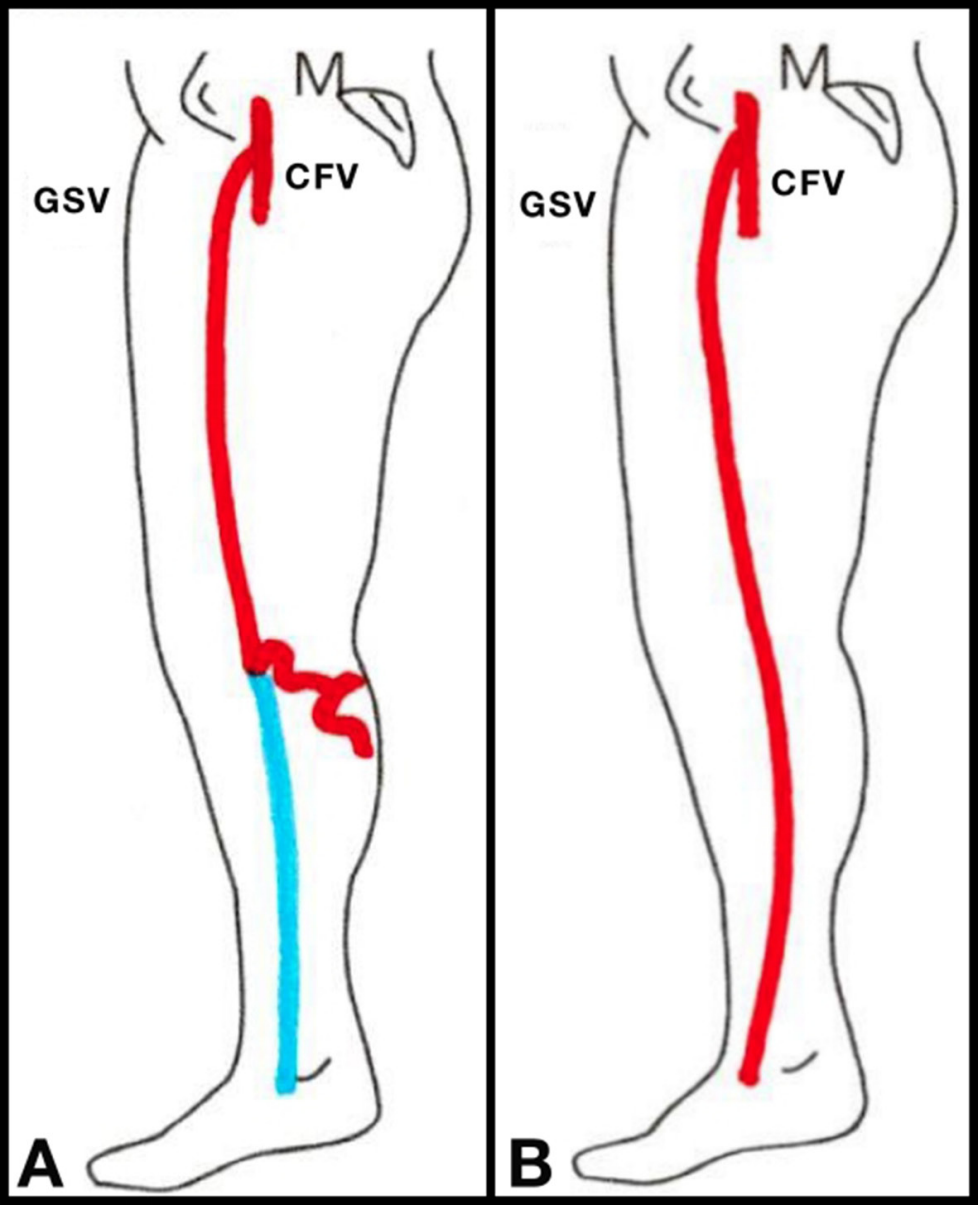
Illustration of a proximal reflux pattern in the control group (A) and a diffuse reflux pattern in the test group (B). CFV, common femoral vein; GSV, great saphenous vein. (Adapted from Engelhorn & Engelhorn^7^).

Participants underwent preoperative clinical evaluation and DUS examination of the superficial and deep venous systems and perforating veins. All examinations were performed with the patient in the upright position and the criterion presence of reflux in the GSV and perforating veins was defined as retrograde flow lasting longer than 0.5 s after manual compression and decompression of the distal vein. Patients were then distributed into 2 groups according to preoperative DUS findings and, using the classification proposed by Engelhorn & Engelhorn,^7^ allocated to a control group, for GSVs with a proximal reflux pattern, or a test group, for GSVs with a diffuse reflux pattern (Figure 1).

All patients were admitted on the same day of surgery after an 8-hour fast. Under spinal anesthesia, the GSV was punctured with a 16- or 18-gauge Abocath needle at the middle third or distal part of the thigh or at the knee level, depending on technical difficulties encountered. A conventional bare-tip 600-µm optical fiber connected to a laser device (Quanta System, Solbiate Olona, Province of Varese, Italy) set at a wavelength of 1470 nm was inserted through the needle puncture into the affected vein. The optical fiber was advanced through the vein under ultrasound guidance in the anterograde direction until the inguinal region was reached, and the fiber tip was positioned approximately 2 cm from the SFJ. After positioning of the fiber tip, the patient was placed in the Trendelenburg position for administration of tumescent fluid around the GSV. At room temperature, 0.5 L of tumescent fluid was prepared using 500 mL of 0.9% saline solution and infiltrated into the saphenous space, involving the entire length of the vein to be treated.

Laser energy was delivered by manually pulling the optical fiber under ultrasound guidance in a distal direction until the end of the desired length of the vein to be treated; no automatic pull-back device was used. The optical fiber was then withdrawn through the needle puncture. The total energy used was recorded as the sum of energy delivered per linear centimeter and used to calculate the mean linear endovenous energy density (LEED, in J/cm) that was required to achieve occlusion of the saphenous vein treated.

The following additional surgical procedures were also performed with the patient in the Trendelenburg position: phlebectomies of varicose tributaries and insufficient perforating-communicating veins through mini-incisions and closure of larger incisions with nylon suture (5.0 Mononylon®, Ethicon). Occlusive dressings were applied to insertion sites and semi-compressive dressings with orthopedic stockinette and crepe bandage were applied to lower limbs.

Patients were encouraged to walk after recovery from anesthesia. Analgesics were prescribed for pain relief if necessary and non-steroidal anti-inflammatory drugs for 3 days. Bandages were usually removed on the third postoperative day. Patients were instructed to wear above-knee graduated (20-30 mmHg) compression stockings daily for 7 days and allowed to resume their usual daily activities, avoiding physical activity for 15 days. They were asked to return for a follow-up appointment in 3 to 5 days.

Follow-up DUS was performed at 3-5 days after the procedure and at 1, 6, and 12 months for assessment of above-knee GSV obliteration rate and blood flow pattern in the untreated below-knee segment of the GSV, including investigation of the deep venous system to exclude venous thrombosis. All follow-up examinations were performed by the same experienced sonographer who was blinded to the results of preoperative assessments and to group assignment.

The postoperative DUS blood flow pattern in the below-knee segment of the GSV was classified as follows: normal (absence of reflux); total reflux (reflux > 0.5 s extending throughout the length of the below-knee GSV); proximal segmental reflux (reflux > 0.5 s in the proximal part of the below-knee segment); and distal segmental reflux (reflux > 0.5 s in the distal part of the below-knee segment). At each follow-up visit, possible procedure-related complications were evaluated and treated according to a protocol for postoperative symptoms. Follow-up examinations included clinical evaluation using the venous clinical severity score (VCSS).

Patients with recanalization or failure of the above-knee EVLA and patients who were lost to follow-up were excluded from the final analysis.

Quantitative variables are expressed as mean (SD), median, and minimum and maximum values. Qualitative variables are expressed as frequencies and percentages. For quantitative variables, comparisons between groups were performed using Student’s *t* test for independent samples and the nonparametric Mann-Whitney test. The two groups were compared for the likelihood of having normal flow in the GSV, at each time point, using Fisher’s exact test. The Jarque-Bera test was used to test the normality of distribution of quantitative variables. A *p*-value < 0.05 was considered statistically significant. Data were analyzed using IBM SPSS Statistics, version 20.

## RESULTS

From January 2013 to December 2014, 36 patients were enrolled and underwent the proposed treatment regimen, 5 men (13.88%) and 31 women (86.12%). Mean patient age was 45 years (SD, 10,08 years; minimum, 30 years; maximum, 69 years). Mean body mass index was 27.90 (SD, 21,5; minimum, 21.5; maximum, 38), and mean operating time was 81.16 min (SD, 23,30 min; minimum, 45 min; maximum, 150 min).

A total of 59 GSVs were treated, 28 in the right lower limb (47.45%) and 31 in the left lower limb (52.55%). Of these, 2 limbs were CEAP clinical class C2, 34 were C3, 18 were C4, and 5 limbs were C5. The technique of fiber insertion through the needle puncture was used in all 59 treated GSVs (100%).

**Figure 2:**
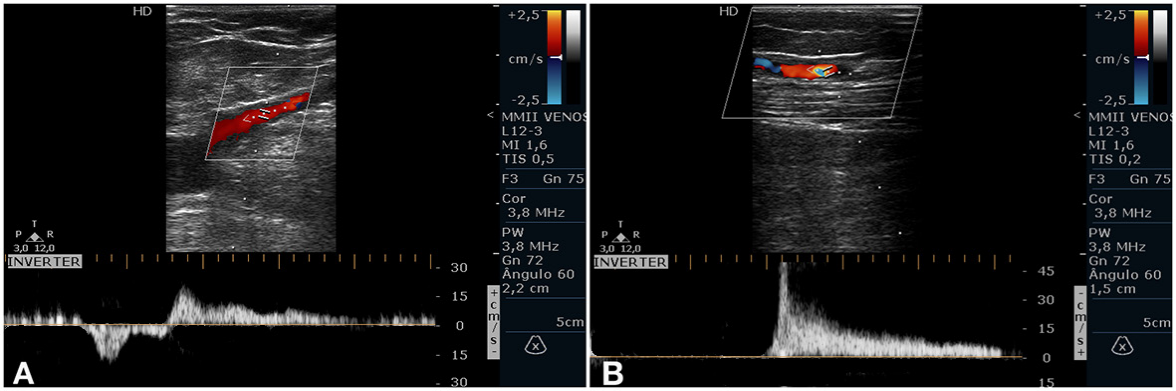
(A) Great saphenous vein (GSV) recanalization at the saphenofemoral junction after 6-month follow-up; (B) Above-knee GSV segmental recanalization after 1-year follow-up.

Based on preoperative findings, 26 GSVs were initially assigned to the control group (normal flow) and 33 GSVs to the test group (below-knee reflux). Of the initial sample of 59 GSVs, 24 GSVs in the control group and 30 GSVs in the test group completed 1-year follow-up and were included in the final analysis. Overall, 5 treated limbs were excluded. Two patients did not attend the scheduled follow-up appointments during the year: one of these patients had had both limbs treated and the other patient had had 1 limb treated, totaling 3 GSVs lost to follow-up. In 2 cases, the above-knee treatment failed and segmental recanalization occurred: 1 GSV was excluded at 6 months and the other GSV at 1 year (Figure 2).

There was no statistically significant difference in CEAP classifications between control and test groups (median of 3 in both groups; *p* = 0.767) or in terms of mean LEED (61.23 J/cm in the control group vs. 60.77 J/cm in the test group; *p* = 0.954).

**Figure 3:**
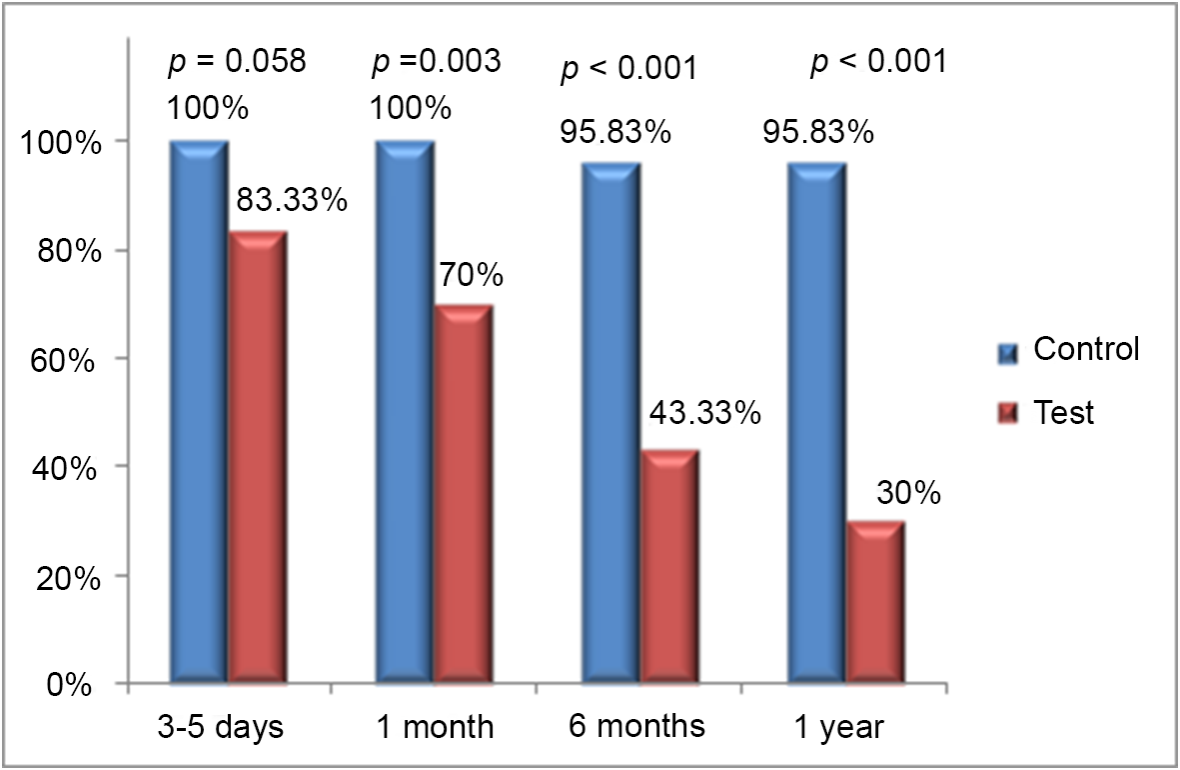
Percentage rate of below-knee great saphenous veins (GSVs) classified as having normal flow at each postoperative time point in the control and test groups.

The DUS examinations of blood flow pattern in the below-knee segment of the GSV at each postoperative assessment time point showed that most patients in the test group had normalization of reflux with normal flow at the beginning of follow-up (88.33% of GSVs at 3-5 days and 70% at 1 month). However, in most of these patients reflux eventually returned (56.67% of GSVs at 6 months and 70% at 1 year) (Figure 3).

**Figure 4:**
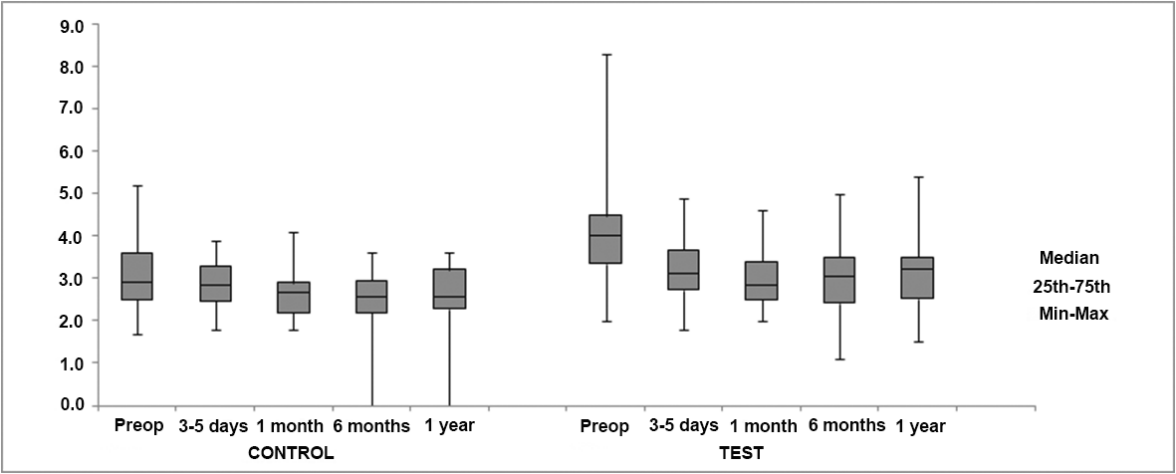
Comparison of changes in great saphenous vein (GSV) diameters (mm) measured below the knee (mid-calf) between the control and test groups.

**Figure 5:**
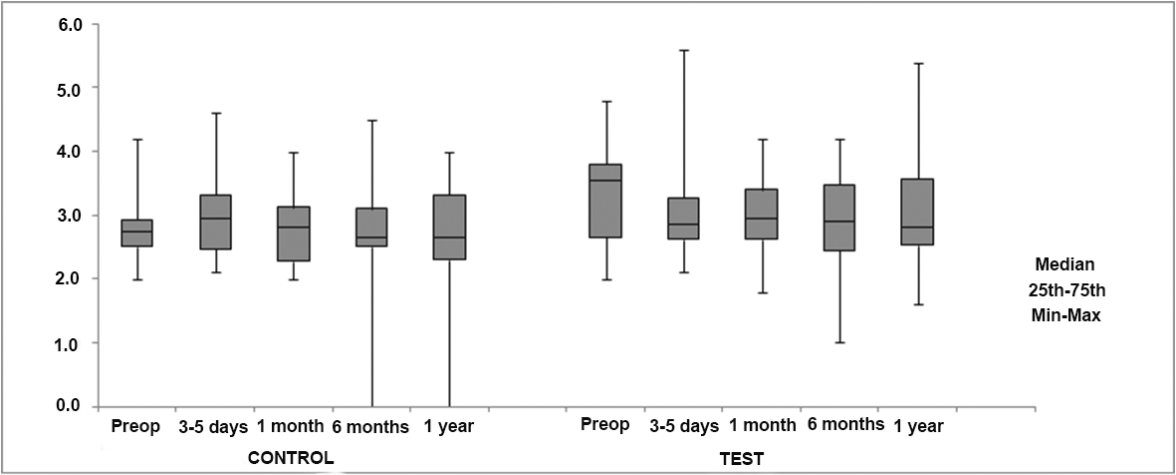
Comparison of changes in great saphenous vein (GSV) diameters (mm) measured at the ankle between the control and test groups.

Regarding changes in GSV diameters measured below the knee (mid-calf and ankle), a statistically significant decrease was observed in mid-calf measurements in the test group (*p* = 0.019) (Table 1). However, no significant differences were found when changes in GSV diameters measured at the mid-calf and ankle were compared between the control and test groups (Figures 4 and 5).

**Table 1:** Changes in great saphenous vein diameter (mm) measured below the knee (mid-calf) in the test group over 1-year follow-up.

**Time of assessment**	***n***	**Mean**	**Median**	**Minimum**	**Maximum**	**SD**	***P*-value** *
Preoperative	30	4.1	4.0	2.0	8.3	1.4	
3-5 days	30	3.3	3.1	1.8	4.9	0.8	
1 month	30	3.0	2.9	2.0	4.6	0.7	0.019
6 months	30	3.0	3.1	1.1	5.0	0.8	
1 year	30	3.1	3.2	1.5	5.4	0.8	

* Nonparametric Friedman test; *p* < 0.05.

**Figure 6:**
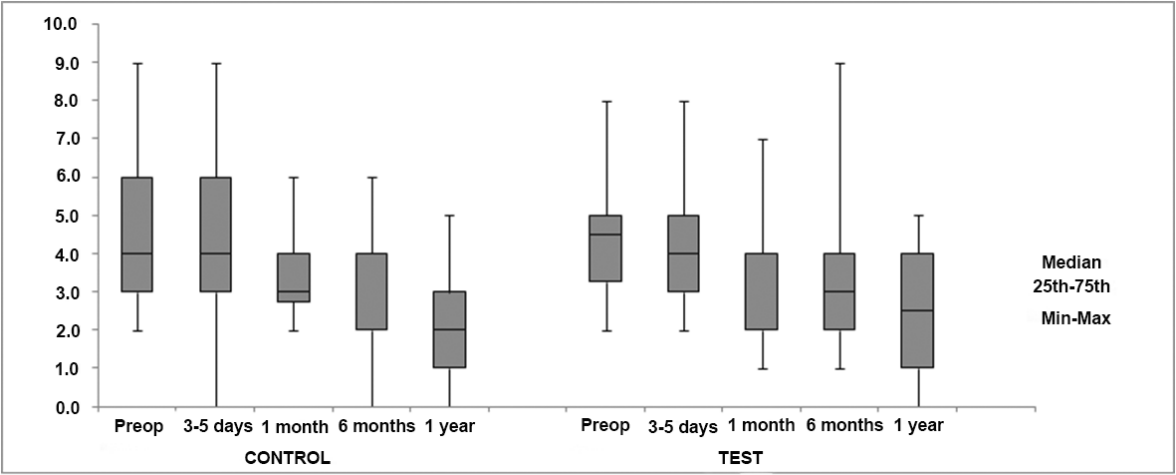
Venous clinical severity score (VCSS) at each postoperative time point in the control and test groups.

The VCSS significantly improved in both groups, but there was no statistically significant difference between groups at any of the time points (Figure 6).

Regarding procedure-related complications, one patient in the test group had thrombophlebitis of the varicose vein at the knee level at 1 month, which resolved spontaneously during follow-up. Two patients in the control group and 1 patient in the test group reported symptoms of paresthesia below the knee in one of their limbs at the first follow-up appointment, which persisted over the first month in 2 of these patients. By 1 year, symptoms resolved spontaneously in all 3 patients.

There was one case of endovenous heat-induced thrombosis with minimal thrombus protrusion through the SFJ into the common femoral vein (involvement of 25% of the vein lumen), and the patient was treated with anticoagulation. The DUS examination was repeated after 4 weeks and showed thrombus regression and resolution, and anticoagulation was then withdrawn.

## DISCUSSION

In most patients DUS-based follow-up of the below-knee segment of the GSV after above-knee EVLA shows that this segment remains patent, although, in rare cases, patients might have occlusion secondary to thrombophlebitis. Therefore, blood continues to be drained from the below-knee GSV to the deep veins through the perforating veins. Persistent below-knee GSV reflux can occur in the presence of incompetent perforating veins or in the presence of a patent tributary in continuity with the proximal untreated GSV, and anterograde flow in this tributary appears to promote continuing reflux. This is also the case when residual varicosities are present in connection with the below-knee GSV.^6^

Pittaluga et al. reported changes in reflux characteristics of the GSV in the short term after isolated phlebectomies with preservation of the GSV, leading to a significant reduction in reflux duration and peak reflux velocity. Isolated phlebectomies also led to a significant decrease in GSV diameter. The observation of a greater decrease in the postoperative diameter of the distal GSV after the phlebectomy with preservation of the GSV could mean that the distal GSV has a greater ability to reduce its diameter after phlebectomy, although that study had a short follow-up period and the same results had not been observed in previous studies.^8^

Opponents of the need for treatment of the refluxing below-knee segment of the GSV suggest that symptoms of venous insufficiency improve after treatment of the above-knee segment, reducing the pressure by excluding a long segment of the incompetent vein. In theory, this would reduce the pressure on the below-knee segment of the GSV and on the varicose veins related to this segment. However, some of these patients return with persistent reflux or worsening of symptoms.^9^

Varicose veins arising from the GSV may communicate with many other vessels. After ablation of the above-knee segment of the GSV, all the varicose veins that are directly connected to this segment tend to decrease in diameter and may disappear. In contrast, varicose veins that are in direct continuity with the untreated below-knee segment of the GSV continue to receive blood from this vein and may persist even after successful above-knee EVLA. Although surgical treatment with phlebectomy or foam sclerotherapy will destroy the residual varicose veins, it seems logical to assume that the requirement for additional treatment may be relaxed if the segment from the SFJ to the most distal point of the incompetent GSV is subjected to EVLA. Thus, elimination of reflux throughout the length of the incompetent GSV should significantly reduce the need for adjuvant therapy for superficial varicose veins, also leading to clinical improvement.^6^

Van Neer et al.^10^ showed that 91% of patients who underwent stripping restricted to the above-knee GSV had persistent reflux of the remaining untreated below-knee segment, indicating that this incompetence of the distal GSV is independent of the proximal GSV segment. Worsening of clinical signs and symptoms occurred between 6 months and 2 years postoperatively, and was accompanied by an increase in reflux and diameters of the below-knee segment of the GSV.

In the present study, although the varicose tributaries and incompetent perforating veins were removed concomitantly with above-knee EVLA of the GSV, an initial improvement in reflux signals (characterized by normal flow in most GSVs) was observed in the test group at the first two follow-up appointments (at 3-5 days and 1 month). However, the initial reflux had returned at 6 and 12 months. Regarding GSV diameters, a significant decrease was observed over time in measurements made at the mid-calf in the test group, although no significant differences were found when GSV diameters measured at the mid-calf and ankle were compared between the two groups.

During EVLA of the GSV, the saphenous nerve is at greatest risk of injury in the mid to distal calf, where it can be injured by direct needle trauma or burned by transfer of energy from the laser, which is an injury that can lead to skin paresthesia, usually transient. Many of these nerve injuries can be prevented by administration of tumescent fluid using ultrasound-guided needle puncture and by avoiding EVLA in areas at high risk of nerve injury.^11^ Limiting the number of tumescent needle punctures and reducing the laser energy along the distal segment of the below-knee GSV may reduce the incidence of paresthesia, but at the expense of decreased treatment success.^12^

Timperman et al. reported that EVLA of the below-knee GSV was highly effective, probably for a number of reasons. First, the diameter of the below-knee GSV is generally smaller than that of the above-knee segment, providing more efficient compression. Second, tumescent fluid can be continuously pumped into the perivenous space during the ablation procedure, ensuring maximal vein compression. Third, high energy can be used to treat this segment (82 J/cm), demonstrating efficacy and safety. Finally, and most importantly, EVLA of the below-knee GSV benefits interruption of the reflux upstream toward the above-knee segment.^12^

Gifford et al. demonstrated efficient occlusion and safe EVLA of the below-knee segment of the GSV with a rate of saphenous neuralgia of 4%, reporting results similar to those found after EVLA of the above-knee GSV alone. The authors concluded that EVLA of the incompetent and symptomatic GSV segment could be considered and performed when other sources of symptoms cannot be confirmed, with excellent ablation and clinical results in the short term.^9^

In conclusion, the results of the present study demonstrate that, although both the control and test groups had a significant improvement in the VCSS and conventional surgical treatment of varicosities and incompetent perforating veins was performed concomitantly with EVLA of the above-knee GSV, most patients in the test group had a return of the reflux at 1 year of follow-up, showing that persistent below-knee GSV incompetence was independent of the treatment performed. Further studies with long-term follow-up are required to determine whether persistent reflux below the knee may influence the recurrence of symptoms.
